# Hair Growth Promotion and Anti-Hair Loss Effects of By-Products Arabica Coffee Pulp Extracts Using Supercritical Fluid Extraction

**DOI:** 10.3390/foods12224116

**Published:** 2023-11-13

**Authors:** Anurak Muangsanguan, Pichchapa Linsaenkart, Tanakarn Chaitep, Jiraporn Sangta, Sarana Rose Sommano, Korawan Sringarm, Chaiwat Arjin, Pornchai Rachtanapun, Kittisak Jantanasakulwong, Yuthana Phimolsiripol, Juan M. Castagnini, Warintorn Ruksiriwanich

**Affiliations:** 1 Department of Pharmaceutical Sciences, Faculty of Pharmacy, Chiang Mai University, Chiang Mai 50200, Thailand; anurak_m@cmu.ac.th (A.M.); pichchapa_li@cmu.ac.th (P.L.); tanakarn_c@cmu.ac.th (T.C.); 2 Master of Science Program in Pharmaceutical Sciences, Faculty of Pharmacy, Chiang Mai University, Chiang Mai 50200, Thailand; 3 Interdisciplinary Program in Biotechnology, Graduate School, Chiang Mai University, Chiang Mai 50200, Thailand; jiraporn_sangta@cmu.ac.th; 4 Cluster of Valorization and Bio-Green Transformation for Translation Research Innovation of Raw Materials and Products, Chiang Mai University, Chiang Mai 50200, Thailand; sarana.s@cmu.ac.th (S.R.S.); korawan.s@cmu.ac.th (K.S.); 5 Center of Excellence in Agro Bio-Circular-Green Industry (Agro BCG), Agro-Industry, Chiang Mai University, Chiang Mai 50100, Thailand; pornchai.r@cmu.ac.th (P.R.); kittisak.jan@cmu.ac.th (K.J.); yuthana.p@cmu.ac.th (Y.P.); 6 Department of Plant and Soil Sciences, Faculty of Agriculture, Chiang Mai University, Chiang Mai 50200, Thailand; 7 Department of Animal and Aquatic Sciences, Faculty of Agriculture, Chiang Mai University, Chiang Mai 50200, Thailand; chaiwat.arjin@cmu.ac.th; 8 School of Agro-Industry, Faculty of Agro-Industry, Chiang Mai University, Chiang Mai 50100, Thailand; 9 Research Group in Innovative Technologies for Sustainable Food (ALISOST), Department of Preventive Medicine and Public Health, Food Science, Toxicology and Forensic Medicine, Faculty of Pharmacy, Universitat de València, Avenida Vicent Andrés Estellés s/n, 46100 Burjassot, Spain; juan.castagnini@uv.es

**Keywords:** androgenetic alopecia, *Coffea arabica*, hair loss treatment, Sonic Hedgehog, Wnt/β-catenin, waste utilization, 5α-reductase inhibition

## Abstract

Coffee has been a common ingredient in many traditional hair loss remedies, but limited scientific evidence supports its use, particularly in coffee pulp. Androgenetic alopecia (AGA) is caused by androgens, inflammation, and oxidative stress. In the present study, supercritical fluid extraction (SFE) was used under various conditions to obtain six coffee pulp extracts. The SFE-4 extract, using 50% (*v*/v) ethanol as a co-solvent at conditions of 100 °C and 500 bars for 30 min, exhibited the highest phenolic, flavonoid, and caffeine contents. Additionally, the SFE-4 extract increased the migration and cell proliferation of HFDPCs (human hair follicle dermal papilla cells), which control hair cycle regulation, and had scavenging effects on ABTS and DPPH radicals. Additionally, the SFE-4 extract showed potassium ion channel opener activity in HFDPCs, as well as a stimulation effect on the enzyme matrix metalloproteinase-2 (MMP-2) (28.53 ± 1.08% of control), which may be related to the vascular endothelial growth factor (*VEGF*) gene upregulation. In human prostate cancer cells (DU-145) and HFDPC cells, the SFE-4 extract significantly decreased the expression of *SRD5A1*, *SRD5A2*, and *SRD5A3*, an essential pathway involved in AGA. Hair growth factor genes in the Wnt/-catenin (*CTNNB1*) and Sonic Hedgehog (*SHH*, *SMO*, and *GLI1*) pathways could be significantly activated by the SFE-4 extract. These results imply that employing SFE in coffee pulp extraction could help AGA treatment by preventing hair loss and promoting hair growth pathways. This would help small coffee producers gain economic empowerment and ensure the long-term sustainability of agricultural waste utilization.

## 1. Introduction

Coffee, a well-known processed beverage, was consumed at about 160 million tons per year in 2022 [1] and Arabica coffee (*Coffea arabica* L.) is one of the world’s most popular coffees [2]. However, a million cubic tons of waste are generated annually from coffee production of about 40–45% of the total output [1]. The by-products of the coffee industry are coffee pulps (outer skin, pulp, pectin layer, and parchment), spent coffee grounds, and other waste materials. According to the data, coffee pulp is the predominant residue from various stages of coffee production, accounting for nearly 74% of the total by-product volume [3]. Moreover, coffee pulp is a valuable resource, containing a diverse range of nutrients, such as proteins, carbohydrates, fats, and fibers, as well as antioxidant substances including flavonoids, phenolics, and caffeine which could be applied for cosmetic, pharmaceutical, and nutraceutical applications [4,5]. In addition, caffeine and phenolic compounds in the coffee extract have been reported as anti-hair-loss agents through the activation of hair growth factors, including insulin-like growth factor (IGF-1), vascular endothelial growth factor (VEGF), Wnt/β-catenin (CTNNB1), and Sonic Hedgehog signaling pathways (SHH, SMO, and GLI1), and the inhibition of transforming growth factor (TGF-β1) and 5α-reductases (SRD5A) [5,6]. However, there is no scientific report indicating the efficacy of coffee extract, especially coffee pulps extracted using supercritical fluid extraction (SFE) which could influence hair loss treatment.

An efficient extraction process is essential for obtaining a high-quality extract. SFE has emerged as an attractive method for plant extraction. This alternative extraction method offers several positive advantages over traditional extraction techniques, including shorter extraction times, higher extraction yields, and lower environmental effects due to the use of fewer solvents and fewer oxidation or degradation processes during the extraction [7]. In this study, the parameters of SFE, including temperature, pressures, amount of co-solvent, and reaction time, were optimized to obtain the highest yield of bioactive compounds.

The most common type of hair loss is androgenetic alopecia (AGA), which affects both genders, namely male pattern hair loss (MPHL) and female pattern hair loss (FPHL). The prevalence of androgenetic alopecia (AGA) is expected to rise in elderly people [8,9]. Generally, the main stages of hair follicle regeneration consist of the stages of growth (anagen), regression (catagen), resting (telogen), and shedding (exogen), which are affected by several factors [10]. In fact, the most common factor in AGA is the androgen hormone. Dihydrotestosterone (DHT), the potent androgen synthesized from testosterone by the 5α-reductase enzyme, is associated with hair follicular shrinking and the shortening of the anagen phase [11]. Conversely, VEGF promotes angiogenesis in the anagen phase and increases the supply of nutrients and oxygen-rich blood to the hair follicle [12]. The topical application of minoxidil can stimulate hair growth in both males and females with AGA through the vasodilation effect through the opening of potassium ion channels near the hair follicle [13]. In fact, minoxidil is metabolized to minoxidil sulfate, which activates adenosine tri-phosphate-sensitive potassium (K_ATP_) channels to open potassium ion channels. Minoxidil is a potassium channel opener that hyperpolarizes cell membranes, causing vascular muscle dilation and a consequent increase in blood flow [14]. The opening of potassium ion channels supports the growth of HFDPCs that affect hair follicles and promote hair growth [15]. The Wnt/β-catenin and Sonic Hedgehog pathways are involved in hair follicle cell development and proliferation, the extension of the anagen phase, and accelerating hair renewal [16]. Additionally, collagenases, particularly matrix metalloproteinase 2 (MMP-2), control the remodeling and degradation of extracellular matrix components, which are important for the development of the hair follicle cycle [12]. In fact, MMP-2 can regulate the delivery of growth factors and cytokines into the hair follicle by increasing the release of VEGF. It has been reported that the relationship between the proteolytic function of MMP-2 and the expression of vascular growth factor may contribute to maintaining the hair growth cycle [17].

The effects of coffee beans on hair growth promotion have been extensively studied, whereas there is no scientific research on by-products, coffee pulp, or extracts for hair loss treatment. Moreover, the idea of green technology has become more popular regarding ecologically friendly methods for ingredient processing. Therefore, this study aims to evaluate the potential of coffee pulp extracts from the SFE technique in hair loss treatment and prevention. This study concentrated on evaluating bioactive compounds, antioxidant activities, anti-hair loss, and hair growth promotion activities through the activation of potassium channel opening, the upregulation of *VEGF* via MMP-2, anti-androgenic activities, and the expression of hair growth factor in the HFDPCs.

## 2. Materials and Methods

### 2.1. Chemicals and Reagents

The Folin–Ciocalteu reagent was acquired from Merck (Darmstadt, Germany), while the following substances were obtained from Sigma Chemical (St. Louis, MO, USA): 2,2′-azino-bis (ethylbenzthiazoline-6-sulfonic acid) (ABTS), 2,2-diphenyl-1-picrylhydrazyl (DPPH), epigallocatechin gallate (EGCG), gallic acid, 6-hydroxy-2,5,7,8-tetramethylchroman-2-carboxylic acid (Trolox), sulforhodamine B (SRB), dimethyl sulfoxide (DMSO), tolbutamide (TBT), caffeine, concanavalin A, and L-ascorbic acid. Additionally, dutasteride, finasteride, minoxidil, and purmorphamine were obtained from Wuhan W&Z Biotech (Wuhan, China). For cell culture, the Follicle Dermal Papilla Cell Growth Medium Kits (catalog no. 2502) were purchased from Promo Cell GmbH (Heidelberg, Germany), while Dulbecco’s modified Eagle medium (DMEM) (catalog no. 11965175), fetal bovine serum (FBS) (catalog no. SV30160.02), antibiotic-antimycotic (100X; catalog no.15240062), and penicillin–streptomycin (100X; 10378016) solution were obtained from Gibco Life Technologies (Thermo Fisher Scientific, Waltham, MA, USA). All other chemicals used throughout the study were of analytical grade.

### 2.2. Collection of Extract

The coffee berries were obtained from Khun Changkhian Highland Agricultural Research and Training Station, Faculty of Agriculture, Chiang Mai University, Chiang Mai, Thailand, in January 2022. All samples were collected and stored at the Pharmaceutical and Natural Products Research and Development Unit (PNPRDU) at Chiang Mai University, with a voucher specimen number of PNPRDU65006. Then, the samples were cleaned and prepared using a wet process. Initially, well-ripened coffee (dark red) was chosen. Then, the coffee berries were peeled using a high-speed food processor to separate between the coffee beans and the coffee pulp. The coffee pulp was dried using a direct sun dryer for 2–3 weeks. Afterward, the coffee pulp was further dried in a hot air oven at 100 °C until the moisture content reached a constant level of 4.0–6.0% (*w*/*v*) [8]. For the extraction process, 10 g of the dried coffee pulp was mixed with 20 mL of solvent and loaded into the extraction vessel of the Spe-ed™ SFE-2 system (Applied Separations, Allentown, PA, USA) [18]. The extraction conditions were varied based on the types of co-solvents, the different ratios between sample and co-solvent, extraction time, and pressure, as shown in Table 1. The temperature was fixed at 100 °C in all extraction conditions.

### 2.3. Phytochemical Evaluations

#### 2.3.1. Total Phenolic Content

The Folin–Ciocalteu colorimetric method described by Sunanta et al. [19] was used to determine the total phenolic content of all extracts. Briefly, 30 µL of the extract was reacted with 150 µL of 10% (*v*/*v*) Folin–Ciocalteu reagent and then neutralized with 210 µL of 6% (*w*/*v*) saturated sodium bicarbonate. The mixture was left at 25 °C for 2 h. The absorbance was read at a wavelength of 765 nm using a UV–Vis spectrophotometer (EZ Read 400 Flexi, Biochrom, Cambridge, UK). The calibration standard was plotted from the absorbance of standard gallic acid at concentrations ranging from 10 to 200 mg/mL. Finally, the total phenolic content was calculated from the standard curve of gallic acid and expressed as milligrams of gallic acid equivalents per gram of dry weight (mg GAE/g dry weight). All samples were performed in triplicate.

#### 2.3.2. Total Flavonoid Content

The total flavonoid content was determined using the method described by Nazir et al. [20] with some modifications. In brief, the extract (25 µL) was mixed with 125 µL of distilled water, followed by 7.5 µL of 5.0% (*w*/*v*) NaNO_2_ solution. The mixture was allowed to react at 25 °C for 5 min before adding 15 µL of 10% (*w*/*v*) AlCl_3_·6H_2_O solution. After that, 50 µL of 1 M NaOH and 27.5 µL distilled water were subsequently added. The absorbance was taken at a wavelength of 510 nm using the UV–Vis spectrophotometer. The calibration standard was plotted using the absorbance of standard epigallocatechin gallate at concentrations between 30 and 300 mg/mL. Total flavonoid content was expressed as milligrams of epigallocatechin gallate equivalents per gram of dry weight (mg EGCG/g dry weight). All samples were performed in triplicate.

#### 2.3.3. Quantitative Analysis of Phenolic Profiles and Caffeine Content by High-Performance Liquid Chromatography Analysis (HPLC)

The extracts were dissolved in 95% (*v*/*v*) ethanol to obtain a final concentration of 1 mg/mL. The caffeine, chlorogenic acid, gallic acid, and catechin gallate contents were identified using high-performance liquid chromatography (HPLC) (Shimadzu, Kyoto, Japan) equipped with an automatic injection (SIL-20ACHT), diode array detection (CTO-20AC), pump (LC-20AD), and automatic control (CBM-20A). The protocol consisted of a previous study described by Sangta et al. [21] with slight modifications. Reverse-phase column chromatography was prepared with Ultra Aqueous C18 (250 × 4.6 mm, 5 µm) (RESTEK, Bellefonte, PA, USA). The mobile phase consisted of mixtures A and B, where mixture A contained formic acid and distilled water in a 50:50 ratio, while mixture B comprised acetonitrile (ACN), formic acid, and distilled water in an 85:50:10 ratio. The injection volume was 10 µL, and the gradient elution began at a flow rate of 1 mL/min. The starting condition was 80% A in 4 min, then reduced to 25% A in 8 min, held at 25% A for 2 min, raised to 70% A in 3 min, and then increased again to 95% A in 1 min. The overall runtime was 18 min.

#### 2.3.4. Determination of Antioxidant Activities

##### DPPH Radical Scavenging Assay

The DPPH radical-scavenging activity was assessed as previously described by Ruksiriwanich et al. [22]. In short, the extract was added to a 96-well plate, along with 50 µL of distilled water and 50 µL of 0.1 mM ethanolic DPPH solution. The plate was then incubated in the dark at 25 °C for 30 min. The absorbance of the sample was measured at a wavelength of 515 nm using the UV–Vis spectrophotometer. Trolox was used as a standard scavenger. Ethanol was used as a blank in this assay. The DPPH radical scavenging activity was calculated using the Equation (1).

(1) $$DPPH\;radical\;scavenging\;activity\;\left(\%\right)=\frac{\left({Abs}_{control}-{Abs}_{sample}\right)}{{Abs}_{sample}}\times 100$$

where ${Abs}_{sample}$ is the absorbance of the DPPH radical solution mixed with the extract, ${Abs}_{control}$ is the absorbance of the DPPH solution mixed with ethanol.

##### ABTS Radical Scavenging Assay

The ABTS assay was conducted following the method described by Adedapo et al. [23] with minor modifications. The stock solution was prepared by mixing 7.0 mM ABTS solution and 2.45 mM potassium persulfate solution. The ABTS stock solution was then left at 25 °C for 12–16 h. The ABTS stock solution was diluted with ethanol until it reached an absorbance of 0.70 ± 0.02 units at a wavelength of 734 nm. After that, 10 µL of the extract and 200 µL of the ABTS working solution were added to 96-well plates, and incubated in the dark at 25 °C for 30 min. The absorbance of the sample was measured at a wavelength of 734 nm. The calibration standard was plotted with concentrations ranging from 0.001 to 1 mg/mL of Trolox. The ABTS radical scavenging capacity of the extract was calculated using the following Equation (2):

(2) $$ABTS\;radical\;scavenging\;activity\;(\%)=\frac{\left({Abs}_{control}-{Abs}_{sample}\right)}{{Abs}_{sample}}\times 100$$

where ${Abs}_{sample}$ is the absorbance of the ABTS radial mixed with extract, ${Abs}_{control}$ is the absorbance of the ABTS solution mixed with ethanol.

### 2.4. In Vitro Cell Viability and Proliferation Assay

Human hair follicle dermal papilla cells (HFDPCs) were obtained from Promo Cell GmbH (Heidelberg, Germany) and cultured using Follicle Dermal Papilla Cell Growth Medium Kits, supplemented with 1% antibiotic-antimycotic (100×) solution. The immortalized human fibroblast cell line (OUMS-36T-4F; NIBIOHN, JCRB Cell Bank, Ibaraki Osaka, Japan) and DU-145 human prostate cancer cells (American Type Culture Collection, Rockville, MD, USA) were cultured in DMEM supplemented with 10% FBS and 1% penicillin–streptomycin (100×) solution.

The sulphorhodamine B (SRB) assay was conducted to determine the cell viability of HFDPC, human fibroblast, and DU-145 cells, as previously described by Khantham et al. [24] with some modifications. In brief, HFDPC and human fibroblast cells were seeded at a concentration of 2 × 10^5^ cells/mL, while DU-145 cells were seeded at a concentration of 1 × 10^5^ cells/mL in a 96-well plate and incubated for 24 h at 37 °C with 5% CO_2_. After that, the cell was exposed to six serial concentrations (0.0625–2 mg/mL) of each extract and the standard control (TBT, caffeine, concanavalin A, L-ascorbic acid, dutasteride, finasteride, minoxidil, and purmorphamine). The control cells were treated with 0.0625–2% (*v*/*v*) ethanol in an incomplete medium, while an incomplete medium was used as a blank. The cells were exposed to all treatments for 24 h. After incubation, the adherent cells were fixed with 50% (*w*/*v*) trichloroacetic acid for 60 min at 4 °C and stained with 0.04% (*w*/*v*) SRB solution for 30 min. Then, the unbound dye wash was washed with 1% (*v*/*v*) acetic acid and dried. The bound dye was solubilized in 10 mM Tris base and the absorbance was measured at 515 nm. All samples were analyzed in triplicate. The optimal concentration that provided more than 80% cell survival was selected for further studies. The percentage of cell viability was calculated using Equation (3).

(3) $$Cell\;viability\;(\%)=\frac{\left({Abs}_{sample}-{Abs}_{blank}\right)}{\left({Abs}_{control}-{Abs}_{blank}\right)}\times 100$$

where ${Abs}_{sample}$ is the absorbance of the cells treated with coffee pulp extract or the standard treatment, where ${Abs}_{control}$ is the absorbance of the cells treated with ethanol in incomplete medium, and where ${Abs}_{blank}$ is the absorbance of the culture plate.

### 2.5. Cell Migration Assay

The migration capacity of HFDPCs was performed using the scratch assay, as previously described [22] with some modifications. HFDPCs at a concentration of 2 × 10^5^ cells/mL were plated in a 96-well plate and incubated for 24 h. The cells were washed with 1X phosphate-buffered saline solution (1X PBS, pH7.2–7.4). Then, a cell-free zone was generated using a pipette tip. The cell debris was washed three times with 1X PBS and replaced with the extracts or standard control (caffeine, minoxidil, and purmorphamine). The cell migration area was observed at different times at 0, 24, and 48 h after treatment using an inverted microscope. The images were analyzed using ImageJ software version 1.53t (NIH, Bethesda, MD, USA). All samples were performed in triplicate. The percentage of cell migration was calculated by Equation (4).

(4) $$Migration\;(\%)=\frac{\left({Area}_{initial}-{Area}_{at\;specified\;time}\right)}{\left({Area}_{initial}\right)}\times 100$$

### 2.6. Cell-Based Potassium Ion Channel Assay

The potassium ion channel assay was used to determine the effect of potassium ion channel opening on the cell viability of HFDPCs. This study was evaluated according to the previously described protocol [25] with some modifications. In brief, HFDPCs (2 × 10^5^ cells/mL) were plated in a 96-well plate and incubated for 24 h. After that, the cells were pre-exposed with 2.5 mM TBT, a potassium ion channel blocker, for 1 h, followed by sample treatment at a concentration of 0.125 mg/mL for another 24 h. After incubation, the cells were fixed with 50% (*w*/*v*) trichloroacetic acid and stained with 0.04% (*w*/*v*) SRB. The bound dye was solubilized in 10 mM Tris base and measured at 515 nm. All samples were performed in triplicate. 

### 2.7. MMP-2 Activity Assay by Gelatinolytic Zymography

The extracts were tested for the gelatinolytic activity of MMP-2 [26]. In this assay, human fibroblast cells at a concentration of 2 × 10^5^ cells/mL were plated in a 6-well plate and incubated for 24 h. After that, the cells were treated with samples at a concentration of 0.125 mg/mL for 48 h. After treatment, the culture media was collected to determine the gelatinolytic activities of MMP-2 using sodium dodecyl sulfate-polyacrylamide gel electrophoresis (SDS-PAGE). In brief, 10 µL of the cell supernatant was resuspended in loading buffer (0.125 M Tris-HCl pH 6.8, 4% SDS, and 0.04% bromophenol blue) and run on 10% SDS-PAGE containing 0.1% (*v*/*v*) gelatin solution. Then, SDS was removed from the gel. The gel was incubated for 1 h at 37 °C in the renaturing buffer (50 mM Tris, 5 mM CaCl_2_, 2.5% Triton X-100, and 0.02% NaN_3_), followed by the developing buffer (50 mM Tris pH 7.5, 5 mM CaCl_2_, 1% Triton X-100, and 0.02% NaN_3_) for 15–18 h at 37 °C. After that, the gel was dyed with 0.5% Coomassie Brilliant Blue G-250 and de-stained with a washing buffer composed of acetic acid, methanol, and distilled water in a ratio of 10:30:60. Gelatinase activity was observed as a white band against a gray background. The gel was scanned using a gel documentation system (Gel Doc™ EZ Gel, Bio-Rad Laboratories, Hercules, CA, USA) and analyzed by Image LabTM software 5.1 (Bio-Rad Laboratories, USA). All samples were performed in triplicate. Concanavalin A, caffeine, and L-ascorbic acid were used as standard treatments. The MMP-2 activity was calculated using Equation (5).

(5) $$MMP-2\;activity\;(\%)=\frac{\left({Volume\;intensity}_{sample}\right)}{\left({Volume\;intensity}_{control}\right)}\times 100$$

### 2.8. Semi-Quantitative Reverse Transcription and Polymerase Chain Reaction Analysis

Gene expression levels were evaluated by semi-quantitative RT-PCR based on a previous method [27] with some modifications. In brief, DU-145 and HFDPC cells were used to assess hair growth promotion through the inhibition of genes associated with 5α-reductase enzymes (*SRD5A1*, *SRD5A2*, and *SRD5A3*) and the stimulation of genes associated with Wnt/β-catenin (*CTNNB1*), Sonic Hedgehog (*SHH*, *SMO*, and *GLI1*), and angiogenesis pathways (*VEGF*). RNA extraction was performed using the E.Z.N.A.^®^ Total RNA Kit I (Omega BioTek, Norcross, GA, USA). The concentration of total RNA was determined using the Qubit^TM^ RNA HS Assay Kit (Invitrogen, Carlsbad, CA, USA) and the Qubit^TM^ 4 fluorometer (Invitrogen, Carlsbad, CA, USA). The RNA elution was stored at -70 °C until further analysis. Gene expression levels were analyzed using the semi-quantitative RT-PCR method. The synthesis of cDNA was performed using the MyTaq^TM^ One-Step RT-PCR Kit (Bioline, Memphis, TN, USA). The primer sequences are shown in Table 2. The expression of each gene in the treated cells was compared to the expression of the internal control gene (*GAPDH*) in the vehicle-treated control. The results were reported as fold changes in gene expression. In this experiment, the coffee pulp extracts were compared to standard controls (caffeine, minoxidil, purmorphamine, finasteride, and dutasteride) at a concentration of 0.125 mg/mL.

### 2.9. Statistical Analysis

The data are presented as the mean ± standard deviation (SD). Statistical analysis was conducted using SPSS 23.0 Software (SPSS Inc., Chicago, IL, USA) with a one-way ANOVA followed by Tukey’s test. Statistical significance was determined as a *p*-value below 0.05.

## 3. Results and Discussion

### 3.1. Extraction Process

The coffee pulp extracts have a greasy, dark gray, semisolid texture. The extraction yields varied between 0.70 ± 0.03% and 16.40 ± 0.82% (*w*/*w*) based on the dry coffee pulp weight (Table 3). Among all extracts, the SFE-6 extract (water as a co-solvent) showed the highest extraction yield, followed by the SFE-5 extract (25% (*v*/*v*) ethanol as a co-solvent) and the SFE-4 extract (50% (*v*/*v*) ethanol as a co-solvent) of 16.40 ± 0.28%, 11.50 ± 0.66%, and 8.60 ± 0.13%, respectively. The extraction conditions for SFE-6, SEF-5, and SFE-4 were performed at a fixed sample-to-solvent ratio of 1:2, a fixed extraction time of 30 min, and a constant pressure of 500 bar.

These findings highlighted the effects of the utilization of a co-solvent in the extraction system and types of co-solvent on the extraction yields, providing valuable insights for optimizing the extraction process and achieving desired yields for various applications. As shown, the SFE-2 extract (95% (*v*/*v*) ethanol as a co-solvent) gave a higher extraction yield than the SFE-1 extract (no co-solvent) at the same extraction condition with extraction yields of 6.15 ± 0.63% and 0.70 ± 0.14%, respectively. The utilization of co-solvents, such as water, ethanol, and methanol, in the SFE process could increase the extraction yield, consistent with a previous study showing that the extraction yields of SFE with ethanol as a co-solvent are higher than those of a pure SFE system [28]. This study showed a significant difference (*p* > 0.05) in the yields obtained from different co-solvents. The SFE with water as a co-solvent provided the highest yield of extraction, which complies with a previous report that indicated the utilization of water in the SFE exhibited a higher extraction yield than that of ethanol as a co-solvent [29]. Accordingly, the presence of hydroxyl groups in ethanol and water enables the formation of hydrogen bonds with the solute [29] and the higher polarity and shorter chain of water can increase the solubility of polar and semi-polar solutes in the SFE system. 

The effect of pressure on the extraction yield was illustrated in SFE-3 (300 bar) and SFE-4 (500 bar) which used a fixed extraction ratio of sample to solvent (1:2), time (30 min), temperature (100 °C), and the co-solvent of 50% *(v*/*v)* ethanol. The result revealed that increasing the pressure from 300 to 500 bar could enhance the extraction yield from 5.70 ± 0.95% to 8.60 ± 0.13%. Normally, pressure has an impact on the density of carbon dioxide. Increasing pressure during the extraction process results in a higher density of carbon dioxide inside the extraction vessel. Moreover, the higher density of carbon dioxide could increase the permeation of carbon dioxide and the co-solvent through the plant sample, resulting in a higher extraction performance and higher extraction yields [30].

### 3.2. Bioactive Constituents and Antioxidant Activities of Coffee Pulp Extracts

The bioactive compounds in coffee pulp extracts including phenolic compounds, flavonoid compounds, and caffeine were reported in the previous study [21]. In this study, the phenolic and flavonoid compounds of all samples ranged between 0.10–5.78 mg GAE/g dry weight and 0.05–7.43 mg EGCG/g dry weight, respectively. The SFE-4 extract showed the highest contents of total phenolic, total flavonoid, and caffeine contents at 5.78 ± 0.03 mg GAE/g dry weight, 7.43 ± 0.13 mg EGCG/g weight, and 19.49 ± 1.04 mg caffeine/g dry weight, respectively (Table 3). Moreover, in phenolic profiles, SFE-5 provided the highest chlorogenic acid (0.55 ± 0.01 mg of CGA/g dry weight), gallic acid (0.31 ± 0.01 mg GAE/g dry weight), and catechin gallate (0.26 ± 0.01 mg CG/g dry weight), followed by SFE-4 and SFE-6, respectively. Accordingly, the efficient extraction of these compounds from plant material depends on various factors, including the types of co-solvents, extraction temperatures, and mechanical agitation, resulting in a higher concentration of polyphenols in the extracts [31]. Phenolic and flavonoid compounds are secondary metabolites belonging to the polyphenol family [32]. Previous studies reported that coffee pulp extracts were rich sources of flavonoids and phenolics [21]. It is well established that the polarity of the co-solvent has a significant effect on phenolic compound extraction [33]. The chlorogenic acid, gallic acid, and catechin gallate, semi-polar compounds, could be extracted by the SFE technique with ethanol as a co-solvent. These semi-polar biological compounds have been reported to have anti-hair loss activity via anti-androgen pathways by inhibition of the gene expression of 5α-reductase enzymes and to promote hair growth via the stimulation of the expression level of growth factors in the hair follicle development process [34,35]. 

In our study, the antioxidant activities of coffee pulp extracts were determined using the ABTS and DPPH scavenging assays. The SFE-4 extract provided the greatest antioxidant capabilities against ABTS and DPPH radicals among all extracts, at 56.63 ± 1.13% and 36.49 ± 1.24%, respectively. The quantities of polyphenol compounds may significantly contribute to their promising antioxidant activities [36,37]. Indeed, polyphenol compounds possess potential antioxidant capabilities due to the number of hydroxyl groups in their structures [38]. Thus, the SFE-4 extract not only contained the highest total phenolic and total flavonoid contents, but also provided effective antioxidant effects against ABTS and DPPH radicals. 

For the caffeine contents in the extracts, the SFE-1 extract (no co-solvent) exhibited the highest caffeine content at 864.05 ± 6.95 mg caffeine/g extract. Andrade et al. reported that the extracts obtained from the SFE system presented higher caffeine levels than the extracts obtained from the SFE combined with a co-solvent [37]. Although the SFE-1 extract had the highest caffeine content, it had the lowest extraction yield, polyphenol content, and scavenging activities. Additionally, it was found that the caffeine concentration of the SFE-1 extract dropped by approximately 5.18 mg caffeine/g dry weight when expressed on a dry basis. On the other hand, SFE-4 (50% (*v*/*v*) ethanol as a co-solvent) demonstrated the highest caffeine content of 19.50 mg caffeine/g dry weight, which complies with previous studies showing that the utilization of 50% (*v*/*v*) ethanol yielded the highest caffeine content [39]. Overall, the SFE-4 extract, with a ratio of 1:2, a time of 30 min, a temperature of 100 °C, a pressure of 500 bar, and a co-solvent of 50% (*v*/*v*) ethanol, displayed the highest levels of phenolic compounds, flavonoid compounds, caffeine content, and scavenging activity when compared to the other conditions. 

### 3.3. Effect of Coffee Pulp Extracts on Cell Viability and Proliferation

The cytotoxicity of coffee pulp extracts at concentrations ranging from 0.0625 to 2 mg/mL was tested on HFDPC, DU-145, and human fibroblast cells. According to ISO 10993-5, a percentage of cell survival beyond 80% is considered non-toxic [40]. After 24 h of treatments, all coffee pulp extracts at concentrations of 0.0625–0.250 mg/mL for HFDPCs and 0.0625–0.125 mg/mL for DU-145 and human fibroblast cells showed no cytotoxicity at the comparable level of untreated cells (*p* > 0.01). Therefore, the maximum concentration of 0.125 mg/mL that gave no toxicity to the cells and could maintain cell viability above 80% in all three cell types was selected for further experiments.

Moreover, all coffee pulp extracts (at 0.125 mg/mL) exhibited significantly higher HFDPC cell viability than the standard controls (10% FBS, caffeine, minoxidil, and purmorphamine) (*p* < 0.05). HFDPCs, located at the base of the hair follicle, interact with various other cell types within the follicle, such as epithelial stem cells, matrix cells, and melanocytes, and play an important role in the hair cycle, particularly during the anagen phase. The significant HFDPCs proliferation of the coffee pulp extracts in this study could support the growth of hair follicles, resulting in hair growth promotion [41,42]. 

### 3.4. Effect of Coffee Pulp Extracts on Migration of HFDPCs

**Figure 1:**
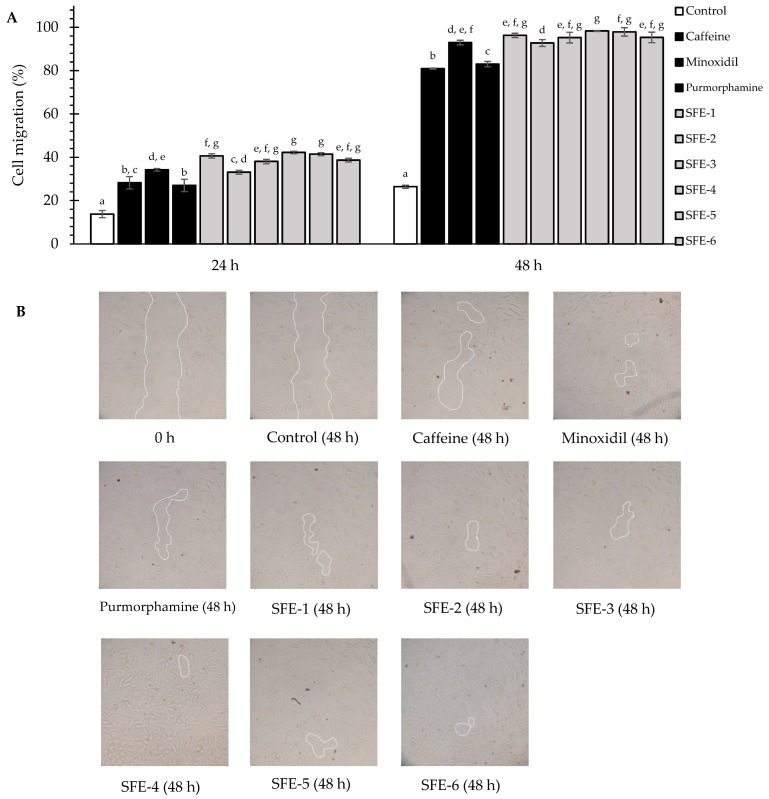
Effects of coffee pulp extracts and standard controls (caffeine, minoxidil, and purmorphamine) at a concentration of 0.125 mg/mL on the migration of HFDPCs using the scratch assay. (**A**) The percentage of migration at each time point was compared to the scratch area at 0 h; (**B**) Microscope images representing HFDPCs’ migration areas at 0 and 48 h after exposure to each sample. Values were expressed as the mean ± SD for triplicates in each group. Statistical analysis was performed using one-way ANOVA, followed by Tukey’s HSD test. Different letters (a–g) indicate statistical differences (*p* < 0.05) in the cell viability of each sample within each treatment duration (24 h and 48 h).

HFDPCs’ migration is crucial in the hair follicle transition process from the telogen to anagen phases. The migration of HFDPCs could enhance dermal papilla volume, hair follicle size, and hair shaft thickness [43]. Figure 1 presents the effects of coffee pulp extracts and standard controls (caffeine, minoxidil, and purmorphamine) on cell migration of HFDPCs. After 48 h, HFDPCs in all treatment groups completed their migration processes with no visible free zone. 

Notably, all coffee pulp extracts demonstrated significantly higher migration effects (*p* < 0.05) than the control at 24 and 48 h and the standard caffeine and purmorphamine at 48 h. The migration of all coffee pulp extracts after 24 and 48 h was in the range of 33.10 ± 1.07% to 42.22 ± 0.73% for 24 h and 92.77 ± 0.95% to 98.33 ± 1.94% for 48 h, respectively, whereas the control values were 13.75 ± 1.66% for 24 h and 26.39 ± 0.75% for 48 h. Furthermore, the SFE-4 extract exhibited a significantly faster migration effect after 24 and 48 h (*p* < 0.05) than those of the control and standard controls. SFE-1, SFE-2, and SFE-3 showed comparable migration effects to those of minoxidil, while SFE-4 showed significantly higher migration effects than standard minoxidil. This finding could be explained by the synergistic effects of compounds present in the SFE-4 extract, including caffeine, polyphenols, and other components, which comply with a previous study showing that caffeine and polyphenols can stimulate cell proliferation and affect migration in hair follicles [25]. However, further investigation of the active ingredient in SFE-4 extract is recommended.

### 3.5. Effect of Coffee Pulp Extracts on Potassium Ion Channel in HFDPCs

**Figure 2:**
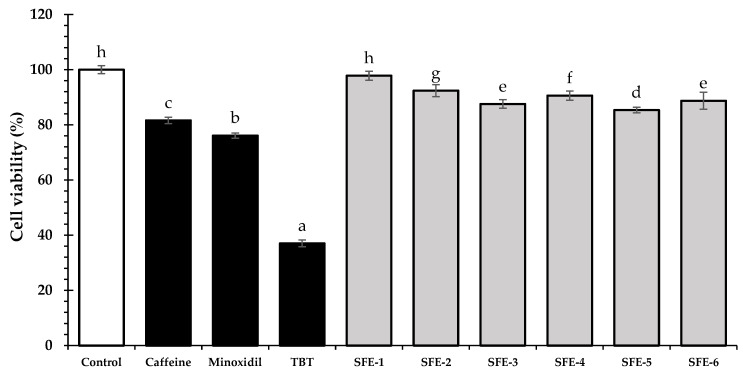
The effect of coffee pulp extracts on potassium ion channels in HFDPCs was compared to the negative control (2.5 mM TBT only) and standard controls such as caffeine and minoxidil. The concentration of SFE extracts was used at 0.125 mg/mL in the SRB assay. In the figure, different letters (a–h) represent statistical differences (*p*-value < 0.05) in the cell viability of each sample according to one-way analysis of variance (ANOVA), followed by Tukey’s HSD test.

It has been reported that potassium ion channels have a significant role in stimulating hair growth, particularly in boosting the growth of HFDPCs by opening potassium ion channels that affect hair follicles and encourage hair growth [44]. It is known that minoxidil, a potent opener of potassium ion channels, acts on various tissues, including vascular smooth muscle to control blood pressure [45] and hair follicles to promote hair growth. In this study, TBT, a potassium ion channel blocker at a concentration of 2.5 mM, was used to block the potassium channel, and then the potassium channel opening ability of the extracts via a cell viability assay was evaluated. As is known, blocking the potassium ion channels causes the mitotic phase, a crucial stage in the cell cycle during the process of cell proliferation, to be disrupted, which results in cell death [46]. The potent potassium channel openers will demonstrate a higher cell viability after the cells are blocked with TBT. TBT showed a hair growth inhibition effect, especially in the anagen stage, by blocking potassium ion channels [44]. The result showed that a negative control (TBT only) significantly decreased the HFDPCs’ cell viability to approximately 37.04 ± 1.21% after 24 h of treatment. In contrast, the coffee pulp extracts exhibited cell viability after cells were blocked by TBT, ranging from 88.74 ± 1.06 to 98.83 ± 1.64%. Among the coffee pulp extracts, the SFE-1 extract showed the highest cell viability of 98.83 ± 1.64%, followed by the SFE-2 extract (92.41 ± 2.15%) and the SFE-4 extract (90.61 ± 1.67%). The SFE-1 extract showed a comparable cell viability to the negative control (without TBT treatment). Furthermore, the cell viability of all coffee pulp extracts was higher than that of caffeine (81.63 ± 1.45%) and minoxidil (76.13 ± 0.95%) (Figure 2). This study indicated that coffee pulp extracts could open the potassium ion channels to an extreme degree after these channels were blocked by TBT, resulting in a higher cell survival. As is known, minoxidil is an antihypertensive vasodilator drug that works by opening potassium ion channels. This drug also exhibited vasodilation at the hair follicle, which increased the blood and oxygen supply and promoted hair growth [13]. Consequently, coffee pulp extracts work through a mechanism similar to caffeine and minoxidil to have a vasodilation impact on HFDPCs.

### 3.6. Effect of Coffee Pulp Extracts on MMP-2 Activity Assay by Gelatinolytic Activity (Zymography)

MMP-2 has been reported to play an important role in the hair cycle, particularly in promoting hair growth by inducing VEGF, a growth factor that stimulates hair follicles [12]. Previous studies have demonstrated that MMP-2 and MMP-9 activate several growth factors, including VEGF, TGF-β1, TNF-α, and IL-1, which are related to hair shaft development [47]. In this study, the impact of coffee pulp extracts and standard controls (concanavalin A, caffeine, and L-ascorbic acid) on MMP-2 gelatinolytic activity was determined by gelatin zymography (Figure 2). Gelatin zymography is a technique that utilizes SDS-PAGE with gelatin to detect gelatinolytic activity in biological products. The appearance of a cleared zone after staining the gel indicates the ability of MMP-2 to degrade gelatin [20]. The highly active MMP-2 activation activity of the extracts from potent VEGF inducers may contribute to a clearer gel band.

**Figure 3:**
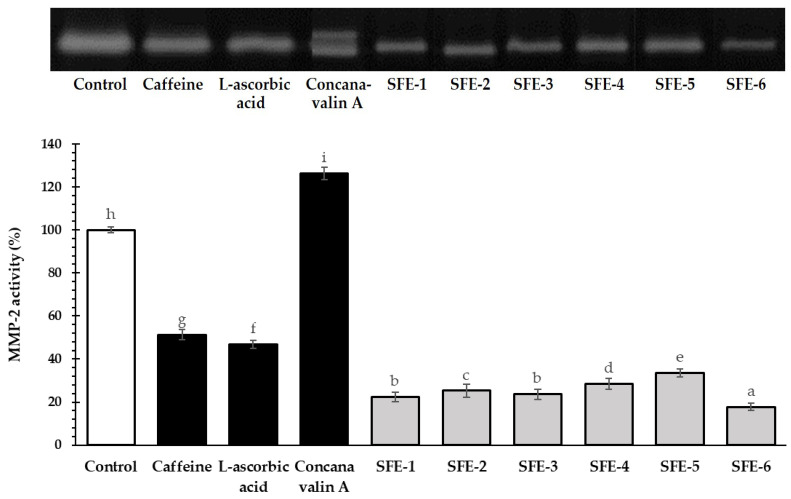
The impact of coffee pulp extracts on MMP-2 expression in human fibroblast cells was assessed at a concentration of 0.125 mg/mL. The standard controls (concanavalin A, caffeine, and L-ascorbic acid) were included for comparison. The results are presented as the percentage of MMP-2 activity relative to the untreated control. Statistical analysis was performed using one-way ANOVA, followed by Tukey’s HSD test. Different letters (a–i) were used to indicate significant differences (*p*-value < 0.05) in the MMP-2 activity of each sample.

The results showed that the MMP-2 activity of all coffee pulp extracts ranged from 17.84 ± 1.02% to 33.53 ± 1.20% (Figure 3). The SFE-5 extract exhibited the highest MMP-2 activity of 33.53 ± 1.20%, followed by the SFE-4 extract (28.53 ± 1.08%) and the SFE-2 extract (25.29 ± 1.22%). However, the stimulation of MMP-2 activity in all coffee pulp extracts was significantly lower than the standard control (concanavalin A at 126.22 ± 2.77%, caffeine at 51.24 ± 1.68%, and L-ascorbic acid at 46.75 ± 1.04%). Interestingly, the percentage of MMP-2 activity within coffee pulp extracts exhibited a correlation with the mRNA expression levels of *VEGF* [12]. This finding is related to previous research indicating that MMP-2 induces growth factors, including VEGF, TGF-β, TNF-α, and IL-1α [12] which showed its ability to promote anti-hair loss and hair growth in human dermal papilla.

### 3.7. Effects of Coffee Pulp Extracts on Genes Expression

**Figure 4:**
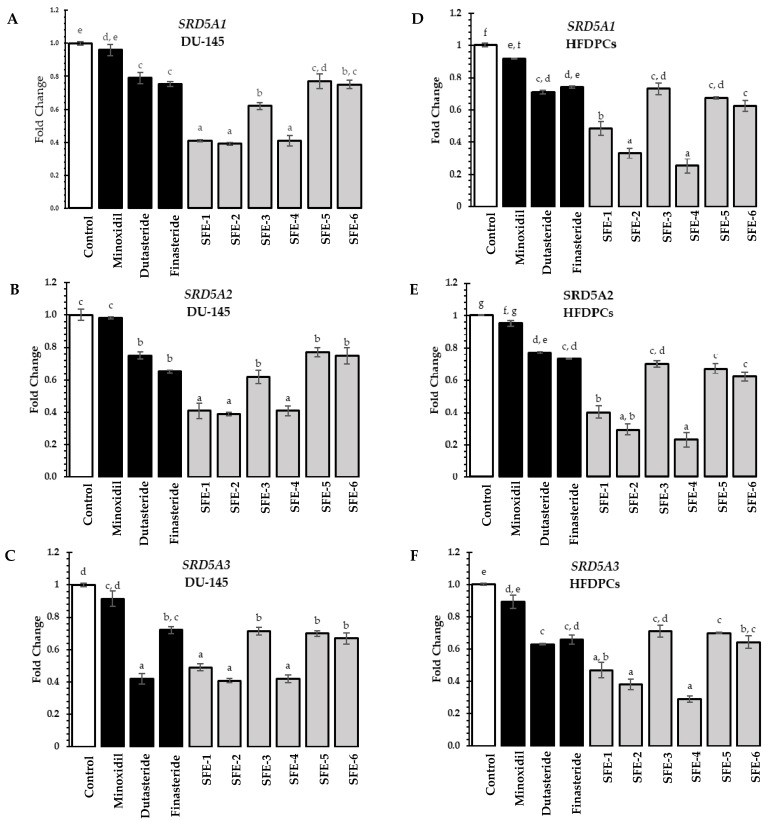
The effects of coffee pulp extracts on the expression of genes in the androgen pathway (**A**) *SRD5A1*; (**B**) *SRD5A2*; and (**C**) *SRD5A3* in DU-145 (**D**) *SRD5A1*; (**E**) *SRD5A2*; and (**F**) *SRD5A3* in HFDPC cells were compared to the standard treatment (minoxidil, dutasteride, and finasteride) at a concentration of 0.125 mg/mL. The results are indicated as a fold change in gene expression relative to the control (untreated). Statistical analysis was performed using one-way ANOVA, followed by Tukey’s HSD test. Different letters (a–g) within each sample indicate significant differences (*p*-value < 0.05).

The key regulatory pathways of hair regeneration, including the androgen pathway, Wnt/β-catenin, Sonic Hedgehog, and angiogenesis signaling pathways, were determined in this study. The 5α-reductase enzymes convert testosterone into DHT, a more potent androgen. The overproduction of DHT may shorten the anagen phase in the hair cycle, resulting in the shrinkage of the hair follicle and eventually hair loss [48]. The 5α-reductase isoenzymes are now the main targets for hair loss treatment [49]. Previous studies reported that unsaturated fatty acids and alpha-tocopherol in rice bran played important roles in 5α-reductase enzyme inhibition [50,51,52]. Initially, we investigated the effects of coffee pulp extracts on the expression of genes encoding *SRD5A1*, *SRD5A2*, and *SRD5A3* associated with the androgen pathway in DU-145 and HFDPC cells. The extracts and standard treatments, including minoxidil, dutasteride, and finasteride, were tested at the same concentration of 0.125 mg/mL. The inhibitory effects of coffee pulp extracts on the genes associated with the androgen pathway are presented in Figure 4. 

In both DU-145 and HFDPC cells, all extracts significantly suppressed the expression of *SRD5A1*, *SRD5A2*, and *SRD5A3* compared to the untreated group (*p <* 0.05). The extracts from SFE-1, SFE-2, and SFE-4 showed the highest *SRD5A1*, *SRD5A2*, and *SRD5A3* gene suppression and significantly higher activities than the standard controls (minoxidil, dutasteride, and finasteride) in both DU-145 and HFDPC cells (*p* < 0.05). For the overall results of the investigation of *SRD5A* gene suppression among DU-145 (the prostate cancer cell lines as a common source of 5α-reductase isoenzymes) and HFDPCs (the origin of the hair shaft and the center of hair growth promotion), the *SRD5A* gene suppression in HFDPCs might have a stronger reliability than DU-145. The SFE-4 extract had the highest fold change in *SRD5A1*, *SRD5A2*, and *SRD5A3* expression in HFDPCs at 0.25 ± 0.04, 0.23 ± 0.03, and 0.29 ± 0.01, respectively. The SFE-4 extract was chosen as having the highest *SRD5A* suppression, since it not only showed the highest levels of *SRD5A* suppression in HFDPCs but also in DU-145 cells due to its high content of bioactive compounds (polyphenols and caffeine) and its promising free radical scavenging activity [53]. This finding is related to previous research indicating that the effects of polyphenols and caffeine involve anti-hair loss by inhibiting the 5α-reductase enzyme [34,54].

The development of hair follicles and the *SRD5A* gene suppression are both important factors in the complicated mechanism of hair growth. The development of hair follicles requires interaction between the surface epithelial cells and the inner mesenchyme through secreted signaling molecules. The activation of hair follicle germs, which are regular thickenings in the epithelial layer caused by a signal from the dermis, is a key stage in this process. The dermal papillae that grow within hair follicles are structurally influenced by the signaling from the hair germs, which in turn drives the clustered creation of underlying HFDPCs. The dermal papilla and hair germs need to communicate effectively in order to control hair germ multiplication and promote hair growth [55]. The complex process of hair formation involves several signaling pathways, including the Wnt/β-catenin, Sonic Hedgehog, and angiogenesis pathways [56]. The Wnt/β-catenin (CTNNB1) signaling pathway is an important mechanism involved in the development of hair follicles and the interfollicular epidermis as a master regulator of hair follicle development. Wnt/β-catenin activity in HFDPCs contributes to the extension of the anagen phase and promotes new hair growth [57,58,59]. The Wnt/β-catenin signaling pathway is connected to the Sonic Hedgehog pathway as an upstream factor of Sonic Hedgehog (SHH, SMO, and GLI1) signaling during the formation of hair follicles [60]. The smoothened (SMO) molecule, the transcription factor, and the glioma-associated oncogene 1 (GLI1) are all activated along the Sonic Hedgehog pathway when the Sonic Hedgehog (SHH) molecule attaches to its receptor [61]. From there, the transcription of the genes responsible for hair growth starts. The anagen phase of the hair follicle is initiated by the Sonic Hedgehog pathway, which controls the proliferation of HFDPCs and stimulates hair follicle development [62]. The shift from the telogen to the anagen stages is supported by the Sonic Hedgehog pathway [63]. In addition, *VEGF* serves as an indicator of angiogenesis during the active growth phase of hair follicles [12]. Its mechanisms involve facilitating the delivery of oxygen and nutrients to the hair follicles, increasing the hair follicles’ diameter, and resulting in hair growth [64].

The regulatory effects on hair follicle development of coffee pulp extracts on the *CTNNB1*, *SHH*, *SMO*, *GLI1*, and *VEGF* genes involved in hair growth pathways were investigated. In HFDPCs, the extracts and standard treatments, including minoxidil and purmorphamine, were tested at the same concentration of 0.125 mg/mL.

**Figure 5:**
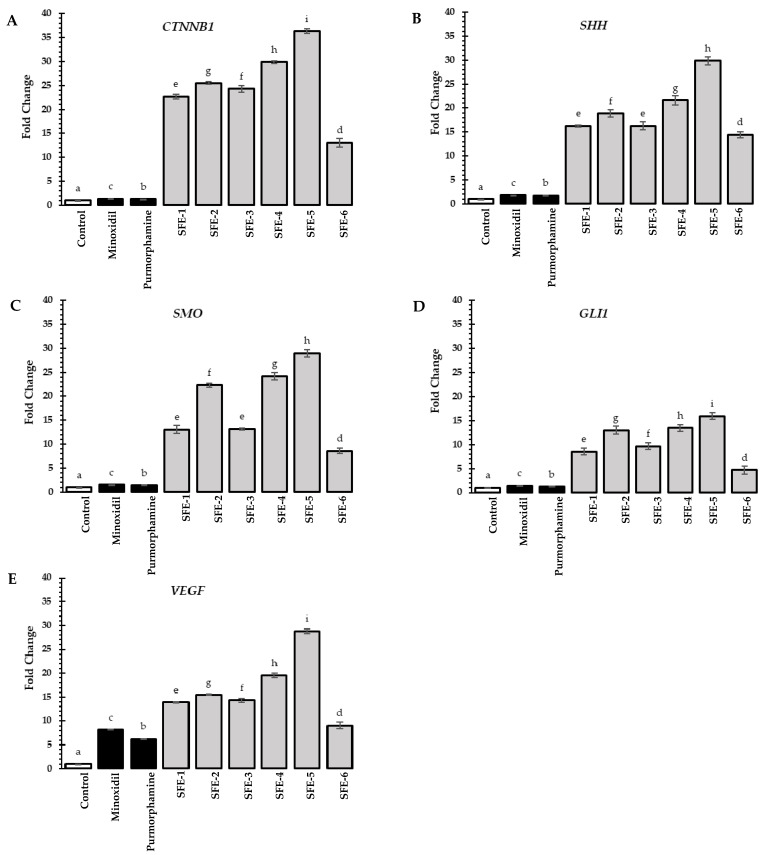
Effects of coffee pulp extracts and standard treatments (minoxidil and purmorphamine) (0.125 mg/mL) on the relative mRNA expression of genes associated with (**A**) Wnt/β-catenin signaling *(CTNNB1)*; (**B**) Sonic Hedgehog pathways (*SHH)*; (**C**) *SMO*; (**D**); *GLI1*; and (**E**) angiogenesis *(VEGF)* in HFDPCs. Statistical analysis was performed using one-way ANOVA, followed by Tukey’s HSD test. Different letters (a–i) above the bars indicate significant differences (*p* < 0.05).

Regarding the Wnt/β-catenin pathway (Figure 5A) and the Sonic Hedgehog pathway (Figure 5B–D), all coffee pulp extracts significantly stimulated *CTNNB1*, *SHH*, *SMO*, and *GLI1* expression compared to the untreated group (*p* < 0.05). Furthermore, all coffee pulp extracts exhibited significantly higher levels of *CTNNB1*, *SHH*, *SMO*, and *GLI1* expression than those of the standard (minoxidil and purmorphamine) treatments (*p* < 0.05). For the Wnt/β-catenin pathway, the SFE-5 extract exhibited the highest fold change in *CTNNB1* expression of 36.32 ± 0.48, followed by the SFE-4 extract (29.89 ± 0.25) and the SFE-2 extract (25.48 ± 0.28), respectively. For the Sonic Hedgehog pathway, the SFE-5 extract exhibited the highest fold change in all tested genes of *SHH* (29.87 ± 0.82), *SMO* (28.92 ± 0.77), and *GLI1* (15.91 ± 0.74), followed by SFE-4 and SFE-2, respectively. 

All coffee pulp extracts remarkably stimulated the levels of *VEGF* expression compared to the untreated group and standard treatment. Notably, SFE-5 exhibited the highest stimulation of *VEGF* expression (28.81 ± 0.53), followed by SFE-4 (19.51 ± 0.52) and SFE-2 (15.50 ± 0.15), respectively. This might be explained by the synergistic actions of other polyphenols in the extract. Previous studies have demonstrated that polyphenols, such as quercetin, caffeic acid, and chlorogenic acid, may enhance *VEGF* expression and accelerate wound healing [65,66]. Additionally, our results showed that coffee pulp extracts outperformed the standard control, especially minoxidil, which is known to stimulate *VEGF* expression and its receptor in HFDPCs, encouraging angiogenesis during the active hair growth phase [67].

In the overall hair growth promotion pathway, the SFE-5 extract exhibits the highest stimulation of all growth factors, followed by the SFE-4 and SFE-2 extracts, respectively. Although the SFE-5 extract has less phenolic compounds, flavonoids, and caffeine than the SFE-4 extract, the SFE-5 extract contains higher amount of chlorogenic acid (0.55 ± 0.02 mg of chlorogenic acid/g dry weight) than the SFE-4 extract (0.42 ± 0.02 mg of CGA/g dry weight). This result corresponds with a previous study that found chlorogenic acid stimulates the expression level of growth factors in hair follicle development [34]. The extraction ratios for the extracts from SFE-5 and SFE-4 were fixed (1:2, 30 min, 100 °C, and 500 bar), but the percentages of co-solvents varied: SFE-5 used 25% *(v*/*v)* ethanol, whereas SFE-4 used 50% *(v*/*v)* ethanol. A co-solvent of SFE-5 with a higher polarity could extract a higher concentration of chlorogenic acid than SFE-4, since chlorogenic acid is a highly polar molecule.

The chosen extract must have a positive impact on both *SRD5A* suppression and growth factor expression to be a potential candidate for anti-hair-loss and hair growth promotion actions for AGA pathogenesis. The SFE-4 extract had promising results on *SRD5A1*, *SRD5A2*, and *SRD5A3* suppression among other extracts. Moreover, the SFE-4 extract contains an amount of caffeine (19.49 ± 1.04 mg caffeine/g dry weight), which has been reported to have anti-hair-loss efficacy [34]. Previous research indicates that caffeine inhibits phosphodiesterase enzymes, which increases intracellular cAMP levels and affects HFDPC cell function and proliferation [6]. Furthermore, caffeine could enhance the hair growth stimulation factors Wnt/β-catenin (CTNNB1), Sonic Hedgehog (SHH, SMO, GLI1), and insulin-like growth factor 1 (IGF-1), which lengthens the active phase duration in the hair follicle [6]. Especially caffeine can inhibit hair loss by the inhibition of the SRD5A enzyme, which converts testosterone to dihydrotestosterone in the hair follicle, [68]. In addition, SFE-4 demonstrated high chlorogenic acid, gallic acid, and catechin gallate levels close to those of SFE-5. Notably, these compounds have been reported to stimulate the expression level of growth factors in hair follicle development [34,35]. Chlorogenic acid has been reported to increase Wnt/β-catenin activity in HFDPCs, contribute to the extension of the anagen phase in the hair cycle, and promote new hair growth [69]. Moreover, gallic acid may also reduce the mRNA expression of TGF-β1, a gene that regulates the resting stage of the hair cycle, in hair dermal papilla cells [70]. The lower expression of TGF-β1 could promote hair growth by the acceleration of hair follicles’ transition from the resting stage to the active stage. Furthermore, catechin gallate was a potent inhibitor of the 5α-reductase enzyme type 1 and 2, which is an enzyme involved in androgen pathways [71].

Both SFE-4 and SFE-5 showed promising hair-growth-promoting properties by increasing the expression of hair growth factor genes (*CTNNB1*, *SHH*, *SMO*, *GLI1*, and *VEGF*) and MMP-2 activity. In addition, the SFE-4 extract performed better than the SFE-5 extract in cell migration and potassium ion channel assays. Finally, the SFE-4 extract was selected as a promising candidate for promoting hair growth and preventing hair loss in this study.

## 4. Conclusions

The hair growth promotion and anti-hair loss activities of six coffee pulp extracts, which were extracted under varying conditions of SFE, were examined. The SFE-4 extract showed the highest levels of phenolic compounds, flavonoids, caffeine content, and scavenging activity compared to others. These compounds could promote the cell proliferation and migration of HFDPCs, particularly during the anagen phase. The SFE-4 extract also stimulated potassium channel opening and MMP-2 expression, which could promote hair growth. In addition, the SFE-4 extract demonstrated a stronger inhibitory effect on *SRD5A* expression than finasteride and dutasteride, which decreased the synthesis of androgenic hormone in hair follicles and showed an anti-hair loss effect. The SFE-4 extract increased the expression of several genes, including *CTNNB1*, *SHH*, *SMO*, *GLI1*, and *VEGF*, to promote hair growth by extending the anagen phase and enhancing blood flow to the hair follicles. Based on our findings, SFE-4 is selected for further in vivo testing in volunteers and for development as a potential hair care product. 

## Figures and Tables

**Table 1:** Extraction conditions of coffee pulp extracts.

No.	Ratio (Sample: Co-Solvent)	Co-Solvent	Time (min)	Pressure (bar)
SFE-1	1:0	-	10	300
SFE-2	1:2	95% (*v*/*v*) Ethanol	10	300
SFE-3	1:2	50% (*v*/*v*) Ethanol	30	300
SFE-4	1:2	50% (*v*/*v*) Ethanol	30	500
SFE-5	1:2	25% (*v*/*v*) Ethanol	30	500
SFE-6	1:2	Distilled water	30	500

**Table 2:** Sequences of gene-specific primers used for semi-quantitative RT-PCR.

Pathway	Gene	Accession Number	Forward Sequence	Reverse Sequence
Sonic Hedgehog	*SHH*	NM_000193.4	AAAAGCTGACCCCTTTAGCC	GCTCCGGTGTTTTCTTCATC
	*SMO*	NM_005631.5	GAAGTGCCCTTGGTTCGGACA	CCGCCAGTCAGCCACGAAT
	*GLI1*	NM_005269.3	GCAGGGAGTGCAGCCAATACAG	GAGCGGCGGCTGACAGTATA
Wnt/β-catenin	*CTNNB1*	NM_001330729.2	CCCACTAATGTCCAGCGTTT	AACCAAGCATTTTCACCAGG
Angiogenesis	*VEGF*	NM_001025366.3	CTACCTCCACCATGCCAAGT	GCGAGTCTGTGTTTTTGCAG
5α-reductase	*SRD5A1*	NM_001047.4	AGCCATTGTGCAGTGTATGC	AGCCTCCCCTTGGTATTTTG
	*SRD5A2*	NM_000348.4	TGAATACCCTGATGGGTGG	CAAGCCACCTTGTGGAATC
	*SRD5A3*	NM_024592.5	TCCTTCTTTGCCCAAACATC	TCCTTCTTTGCCCAAACATC
Internal control	*GAPDH*	NM_001289745.3	GGAAGGTGAAGGTCGGAGTC	CTCAGCCTTGACGGTGCCATG

**Table 3:** Biological compounds and antioxidant activities of coffee pulp extract in each extraction condition.

Extract No.	Extraction Yield (%)	Total Phenolic Content	Total Flavonoid Content	Caffeine Content	Chlorogenic Acid Content	Gallic Acid Content	Catechin Gallate Content	Antioxidant Activities (%)	
		(mg GAE/g Dry Weight)	(mg EGCG/g Dry Weight)	(mg CAF/g Dry Weight)	mg CGA/Dry Weight	mg GAE/g Dry Weight	mg CG/g Dry Weight	ABTS	DPPH
SFE-1	0.70 ± 0.14 ^a^	0.10 ± 0.01 ^a^	0.05 ± 0.01 ^a^	5.18 ± 0.84 ^c^	0.08 ± 0.01 ^b^	0.06 ± 0.01 ^b^	0.08 ± 0.01 ^c^	8.75 ± 0.26 ^a^	9.50 ± 0.96 ^a^
SFE-2	6.15 ± 0.63 ^b^	3.14 ± 0.31 ^b^	2.12 ± 0.12 ^c^	15.13 ± 1.12 ^e^	0.02 ± 0.01 ^a^	0.01 ± 0.01 ^a^	0.02 ± 0.01 ^a^	46.41 ± 2.96 ^c^	34.25 ± 0.22 ^c^
SFE-3	5.70 ± 0.95 ^b^	3.30 ± 0.20 ^b^	1.99 ± 0.13 ^b^	4.07 ± 0.56 ^a^	0.15 ± 0.01 ^c^	0.12 ± 0.01 ^c^	0.06 ± 0.01 ^b^	51.59 ± 2.95 ^d^	34.66 ± 2.17 ^d^
SFE-4	8.60 ± 0.13 ^c^	5.78 ± 0.03 ^c^	7.43 ± 0.13 ^e^	19.49 ± 1.04 ^f^	0.42 ± 0.02 ^e^	0.24 ± 0.01 ^d^	0.12 ± 0.01 ^d^	56.63 ± 1.13 ^e^	36.49 ± 1.24 ^e^
SFE-5	11.50 ± 0.66 ^d^	5.67 ± 0.35 ^c^	2.99 ± 0.11 ^d^	4.91 ± 0.62 ^b^	0.55 ± 0.02 ^f^	0.31 ± 0.02 ^e^	0.26 ± 0.01 ^e^	46.20 ± 2.86 ^c^	33.47 ± 1.87 ^b,c^
SFE-6	16.40 ± 0.28 ^e^	3.27 ± 0.29 ^b^	2.15 ± 0.10 ^c^	8.13 ± 1.01 ^d^	0.18 ± 0.01 ^d^	0.13 ± 0.01 ^c^	0.07 ± 0.01 ^b, c^	44.22 ± 1.52 ^b^	32.35 ± 1.62 ^b^

**Note:** Different letters (a–f) indicate statistical differences (*p*-value < 0.05) in coffee pulp extract yield, total phenolic contents, total flavonoid content, caffeine content, chlorogenic acid content, gallic acid content, catechin gallate content, and antioxidant activities of each sample by one-way ANOVA, followed by Tukey’s HSD test. Milligrams of gallic acid equivalents per gram of extract (mg GAE/g dry weight); milligrams of epigallocatechin gallate equivalents per gram of extract (mg EGCG/g dry weight); milligrams of caffeine per gram of dry weight (mg CAF/g dry weight); milligrams of chlorogenic acid per gram of dry weight (mg CGA/g dry weight); and milligrams of catechin gallate per gram of dry weight (mg CG/g dry weight).

## Data Availability

Data is contained within the supplementary material. The data presented in this study are available on request from the corresponding author.

## Supplementary Materials

The following supporting information can be downloaded at: https://www.mdpi.com/article/10.3390/foods12224116/s1, Table S1: Microscopical images represent HFDPCs migration areas at 0 and 48 h after exposure to all coffee extracts in comparison to the standards.
