# Supplementary material for: Hair Growth Promotion and Anti-Hair Loss Effects of By-Products Arabica Coffee Pulp Extracts Using Supercritical Fluid Extraction
Source: Foods. 2023 Nov 13;12(22):4116. doi: 10.3390/foods12224116 (PMC10670875; doi:10.3390/foods12224116)
Supplement: Supplementary file 1 [file foods-12-04116-s001.zip › foods-2609539-supplementary.pdf]

Table S1: Microscopical images represent HFDPCs migration areas at 0 and 48 h after exposure to all coffee extracts in comparison to the standards.

	**0**	**48**
Control	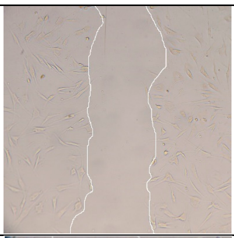	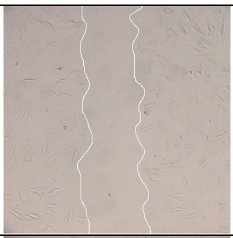
Caffeine	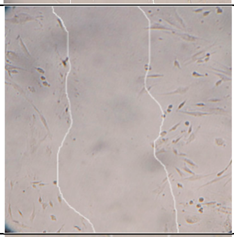	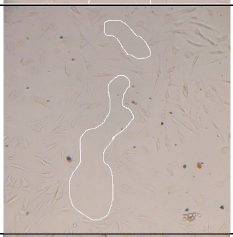
Minoxidil	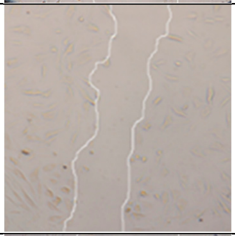	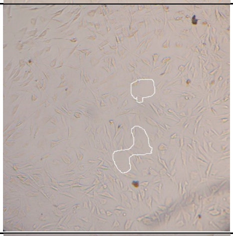
Purmorphamine	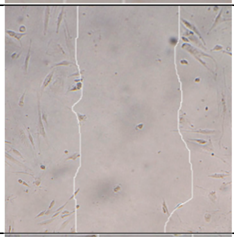	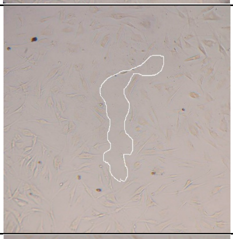
SFE-1	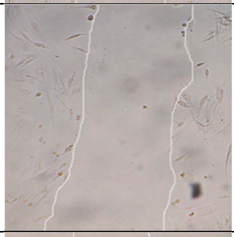	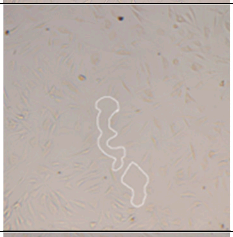
SFE-2	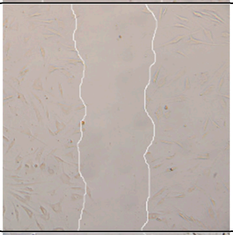	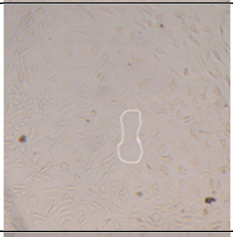
SFE-3	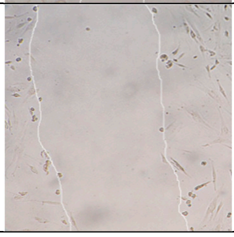	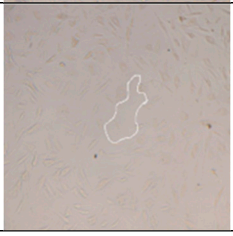

Table 1 Continued: Microscopical images represent HFDPCs migration areas at 0 and 48 h after exposure to all coffee extracts in comparison to the standards.

SFE-4	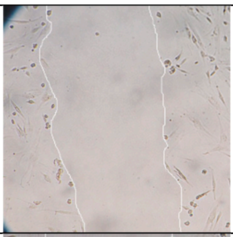	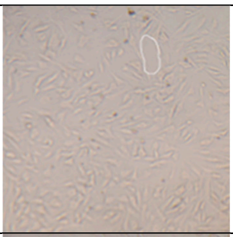
SFE-5	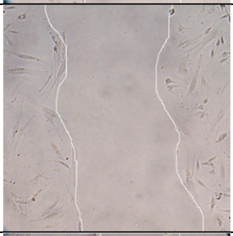	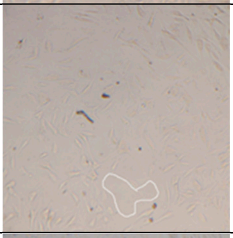
SFE-6	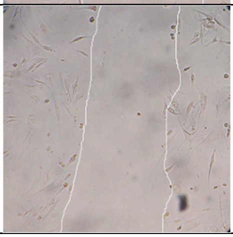	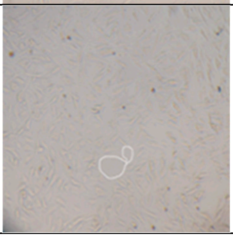
